# Improving Signal-to-Noise Ratio of Drug Fragment Screening with Variational Autoencoder

**DOI:** 10.1063/4.0001064

**Published:** 2025-10-27

**Authors:** Phyllis Zhang, Minhuan Li, Daniel Keedy, Tamar Skaist Mehlman, Doeke Hekstra

**Affiliations:** 1 Harvard College, Cambridge, MA, USA; 2 Harvard University, Cambridge, MA, USA; 3 Flatiron Institute, New York City, NY, USA; 4 CUNY Advanced Science Research Center, New York City, NY, USA

## Abstract

In the quest for new drug candidates, a pivotal phase involves identifying compounds that selectively and robustly bind to their targets to modulate activity for therapeutic effects. This modulation can manifest as inhibition, activation, or allosteric regulation, among others. A core challenge in drug discovery is detecting ligands with high binding affinity to target proteins. Techniques range from high-throughput screening and computational simulations to advanced machine learning models.

Fragment-based drug discovery (FBDD), particularly using X-ray crystallography beamlines, has become a prominent method for finding initial leads for small-molecule modulators. This involves soaking potential ligands into protein crystals, followed by X-ray diffraction data analysis to detect binding fragments. Despite technological advancements enhancing throughput, variations in crystals unrelated to ligand binding hinder analysis sensitivity.

We present VAE-Assisted Ligand Discovery (VALDO), a novel approach to distinguish meaningful conformational changes from unrelated crystal heterogeneity. VALDO employs a variational autoencoder (VAE) to encode crystallographic reflections into a low-dimensional space, filtering out noise and reconstructing an apo state. This facilitates the creation of difference maps crucial for identifying ligand binding. Comparative benchmarks against methods like PanDDA and Cluster4x show VALDO's superior ability to detect and estimate the pose of bound drug fragments.

